# The relationship between novel inflammatory markers SII, SIRI, MHR, UHR and insulin resistance in patients with type 2 diabetes: based on a retrospective analysis

**DOI:** 10.3389/fendo.2025.1648823

**Published:** 2025-09-09

**Authors:** Rongrong He, Hui Sun, Haiying Liu, Jinxia Li

**Affiliations:** ^1^ Clinical Laboratory, Xi’an International Medical Center Hospital, Xi ’an, Shaanxi, China; ^2^ Clinical Laboratory, Xi ‘an Daxing Hospital, Xi ’an, Shaanxi, China; ^3^ Department of Medical Administration and Infectious Disease Supervision, Chang’an District Center for Disease Control and Prevention, Xi ’an, Shaanxi, China

**Keywords:** type 2 diabetes mellitus systemic, systemic immune-inflammation index, systemic inflammation response index, uric acid to HDL-C ratio, monocyte to HDL-C ratio

## Abstract

**Objective:**

This study aims to investigate the relationship between newly identified inflammatory indicators and IR in patients with T2DM, thereby providing a reference basis for the early clinical prevention, diagnosis, and treatment of IR in patients with T2DM.

**Methods:**

A total of 779 patients with T2DM admitted to the Endocrinology Department of our hospital from January 2022 to December 2024 were included in the observation group. Five hundred healthy individuals who underwent physical examinations during the same period were randomly selected as the control group. Patients in the observation group were divided into the IS group, the EIR group, and the SIR according to the HOMA-IR level. Analyze the relationship between the four indicators and IR in patients with T2DM, and observe whether they are independent risk factors for IR in T2DM patients, as well as analyze their clinical utility.

**Results:**

Compared with the control group, the levels of inflammatory indicators SII, SIRI, MHR and UHR in the observation group were significantly increased. The levels of SII, SIRI, MHR and UHR in the EIR group and the SIR Group were significantly higher than those in the IS group. Moreover, with the increase in HOMA-IR score, all four inflammatory indicators showed an upward trend. The results of Spearman’s rank correlation analysis showed that all four indicators were positively correlated with IR in patients with T2DM. Multivariate ordered logistic regression analysis showed that all four indicators were independent risk factors for IR in patients with T2DM. The ROC results indicated that SII, SIRI, MHR and UHR could serve as potential discriminatory ability indicators for evaluating the degree of IR in patients with T2DM.

**Conclusion:**

The levels of SIRI, SII, UHR and MHR in patients with T2DM increase and are positively correlated with IR. They are independent risk factors for IR in patients with T2DM and have clinical utility to a certain extent. They can provide a reference basis for the early clinical prevention, diagnosis and treatment of IR in patients with T2DM.

## 1 Introduction

Diabetes mellitus is a chronic condition commonly encountered in clinical practice, arising from endocrine and metabolic disorders. According to data from the International Diabetes Federation (IDF), approximately 537 million individuals aged 20-79 worldwide were living with diabetes in 2021. This figure is projected to rise to 643 million by 2030, reaching 783 million by 2045 (1, 2). Diabetes can be categorised into type 1 diabetes mellitus (T1DM) and type 2 diabetes mellitus (T2DM) based on differing pathogenic mechanisms, with T2DM accounting for approximately 90% of all cases (3, 4). T2DM primarily develops from unhealthy lifestyle and dietary habits, manifesting characteristic features including insulin resistance (IR), impaired pancreatic β-cell function, and hyperglycaemia (5). Insulin maintains glucose metabolic homeostasis through various physiological responses in tissues such as the liver, skeletal muscle, and adipose tissue. The development of IR leads to glucose metabolism dysregulation, resulting in hyperglycaemia and subsequent diabetes onset. In severe cases, this may progress to multi-organ complications including cardiovascular disease and diabetic nephropathy (6, 7).

The hyperinsulinaemic-euglycaemic clamp (HEC) remains the gold standard for assessing insulin resistance (IR) (8). However, this evaluation method is time-consuming, costly, and technically complex, with limited reproducibility, rendering it unsuitable for routine clinical application (9, 10). The homeostatic model assessment of insulin resistance (HOMA-IR) serves as an alternative approach for IR assessment and is currently regarded as a sensitive indicator for measuring IR (11). HOMA-IR calculations require measurement of fasting plasma insulin levels in patients (11, 12), yet these are not routinely included in standard clinical tests. Consequently, clinicians cannot promptly evaluate IR levels in T2DM patients, necessitating research into simpler, accurate, and cost-effective diagnostic tests to predict IR.

In recent years, epidemiological studies have shown that chronic inflammation plays an important role in the pathogenesis of T2DM (13). Long-term and chronic inflammation in the body leads to the upregulation of inflammatory factors in the islet microenvironment, including interleukin-1 β, CRP, tumor necrosis factor -α, etc., thereby destroying the function and activity of islet β cells and increasing reactive oxygen species, and lead to or aggravate the IR of peripheral tissues (14, 15). Blood components such as neutrophils, lymphocytes, monocytes, and platelets play crucial roles in the development and progression of T2DM (16–18). Serum uric acid (SUA), the end product of dietary and endogenous purine nucleotide metabolism, contributes to atherosclerosis and IR by reducing nitric oxide production, promoting vascular smooth muscle proliferation, and inducing endothelial dysfunction (19). Furthermore, research indicates that low levels of high-density lipoprotein cholesterol (HDL-C) are implicated in the development of metabolic syndrome and IR (20). Therefore, the development of novel biomarkers based on haematological parameters – including complete blood count components (neutrophils, monocytes, platelets, and lymphocytes), SUA, and HDL-C – offers a cost-effective approach to comprehensively assess systemic inflammatory status without imposing additional financial burdens on patients, while facilitating multi-dimensional clinical evaluation. Emerging inflammatory markers derived from these parameters, such as the systemic immune-inflammation index (SII), systemic inflammation response index (SIRI), serum uric acid to HDL-C ratio (UHR), and monocyte to HDL-C Ratio (MHR), have gained widespread clinical application across various pathologies, including diabetes mellitus (21–23).

Whilst associations between novel inflammatory markers (SII, SIRI, UHR, and MHR) and diabetes mellitus have been documented (24–26), their collective significance as determinants of IR in T2DM patients remains unestablished. Concurrently, early identification and clinical intervention of IR demonstrate prognostic value in T2DM management. This study consequently examines the relationship of SII, SIRI, and UHR in T2DM-associated IR, aiming to provide evidence-based insights for the timely prevention, diagnosis, and therapeutic stratification of IR in clinical practice.

## 2 Materials and methods

### 2.1 Study population and data collection

A retrospective analysis was conducted to observe a total of 1589 patients with type 2 diabetes who visited the Department of Endocrinology of Xi ‘an International Medical Center Hospital from January 2022 to December 2024, and they were classified as the observation group. According to the T2DM International Standard (ADA) (27), T2DM is defined as a fasting blood glucose level of ≥ 7.0 mmol/L and/or a 2-hour blood glucose level of ≥11.1 mmol/L and/or a glycated hemoglobin (HbA1c) level of ≥ 6.5% during the oral glucose tolerance test (OGTT). The sample size was estimated using JMP^®^Trial 18.0.1 software (JMP Statistical Discovery LLC., USA). A 95% (α=0.05) confidence interval and 90% (1-β) power were considered to detect a difference of 0.25 units. The calculated sample size is 387. Five hundred healthy individuals who underwent physical examinations during the same period were randomly selected as the control group. Collect the clinical data of the observed subjects, including indicators such as gender, age, BMI, smoking, drinking, blood routine results, and blood lipid results. According to HOMA-IR (28)= (fasting insulin × fasting blood glucose)/22.5, the observation group was divided into the insulin-sensitive (IS) group (HOMA-IR <1.9), the early insulin resistance (EIR) group (1.9≤ HOMA-IR ≤2.9) and the significant insulin resistance (SIR) group (HOMA-IR >2.9) (29).

Inclusion criteria: (1) ≥20 years old; (2) Type 2 diabetes; (3) The clinical data are complete. Exclusion criteria: (1) Type 1 diabetes; (2) Combined with infectious diseases, or malignant tumors, leukemia and other diseases; (3) Those with severe renal insufficiency or anemia and hemolytic diseases; (4) Those who have recently taken drugs that affect uric acid, blood lipids and blood cells; (5) Those with incomplete clinical data. A total of 779 cases were finally included in the observation group.

As this study was a retrospective analysis, informed consent from participants could not be obtained. This study was approved by the ethics committee of our hospital, and its protocol abided by the principles of the Declaration of Helsinki(GJYX-KY-2025-007).

### 2.2 Detect blood and biochemical indicators and calculate the inflammation index

Neutrophils, monocytes, lymphocytes and platelets were counted using the standard automatic hematology analyzer (SYSMEX-XN9000, Japan), and all reagents were provided by the manufacturer. Triglycerides (GPO-PAP method), total cholesterol (CHOD-PAP method, Maccura, China), HDL-C (direct method - peroxidase removal method, Maccura, China), low-density lipoprotein cholesterol (direct method - peroxidase removal method, Maccura, China), SUA (uricase method) and blood glucose (glucose oxidase method, Maccura, China) were measured using an automatic biochemical analyzer (Hitachi - 008as, Japan). The daily laboratory quality control of the above-mentioned projects is under control.

The inflammatory index calculation: SII= neutrophil count (×10^9^/L) ×platelet count (×10^9^/L)/lymphocyte count (×10^9^/L); SIRI =neutrophil count (×10^9^/L) × monocyte count (×10^9^/L)/lymphocyte count (×10^9^/L); UHR = SUA (umol/L)/HDL-C (mmol/L) ratio; MHR =monocyte (×10^9^/L)/HDL-C (mmol/L) ratio;

### 2.3 Statistical analysis

Statistical analysis was conducted using SPSS 22.0 software. Non-normal distribution data were expressed as the median and interquartile range [M (Q3-Q1)], and categorical variables were expressed as percentages. The comparison of non-normal distribution quantitative variables between the two groups was conducted using the two-sample non-parametric test. Non-parametric tests were used for the comparison of non-normal distribution data among multiple groups. The comparison of categorical variable rates was conducted using the chi-square test. The correlations between the four inflammatory indicators and the HOMA-IR score were analyzed using Spearman’s rank correlation analysis. Since the outcome variable (degree of IR) of this study was an ordered multicategorical variable, multivariate ordered logistic regression was used to analyze its influencing factors. The discriminatory ability of four inflammatory indicators for IR of T2DM was evaluated through the ROC curve.

## 3 Results

### 3.1 Comparison of clinical characteristics

#### 3.1.1 Comparison of clinical characteristics and indicators between the observation group and the control group

A total of 500 healthy subjects (control group) and 779 patients with T2DM (observation group) were included. The age distribution of the control group was 56 (51, 60) years old, among which 66.00% were male. The age distribution of the T2DM group was 58 (47, 66) years old, among which 68.42% were male. There was no statistically significant difference in age and gender between the T2DM group and the control group (*P* > 0.05). Compared with the control group, the total amounts of WBC, MONO, PLT, NEUT, TG, UA and glucose in the blood of the T2DM group were significantly higher than those of the control group, and the total amount of LYMP was significantly lower than that of the control group (*P*<0.05). The contents of LDL and HDL in the blood of the T2DM group were significantly lower than those of the control group (*P*<0.05). The values of inflammation indices SII, SIRI, MHR and UHR in the T2DM group were significantly higher than those in the control group (*P*<0.05). As shown in **Table 1**.

**Table 1:** Demographic data and clinical characteristics of control and T2DM patients [(*x* ± *s*), M (Q3–Q1)].

Characteristics	Control (n=500)	T2DM (n=779)	*χ^2^/Z*	*P*
Age (years)	56 (51,60)	58 (47,66)	-1.840	0.066
Male, n (%)	330 (66.00%)	533 (68.42%)	0.209	0.648
Blood routine examination				
WBC	5.53 (5.08,6.49)	5.89 (4.95,7.00)	-2.857	0.004
MONO (×10^9^/L)	0.34 (0.26,0.44)	0.37 (0.30,0.46)	-4.719	0.000
LYMP (×10^9^/L)	1.78 (1.57,2.17)	1.64 (1.36, 2.04)	-5.438	0.000
PLT (×109/L)	198.00 (163.50,220.00)	211.00 (184.00,246.00)	-7.765	0.000
NEUT (×10^9^/L)	3.09 (2.66,3.96)	3.58 (2.83,4.46)	-5.769	0.000
Biochemical indicators				
LDL-C (mmol/L)	2.88 (2.42,3.38)	2.61 (1.91,3.25)	-6.619	0.000
HDL-C (mmol/L)	1.21 (1.08,1.47)	1.02 (0.85,1.23)	-12.387	0.000
TG (mmol/L)	1.01 (0.77,1.63)	1.60 (1.11, 2.48)	-11.648	0.000
TC (mmol/L)	4.63 (4.44, 5.26)	4.67 (3.87, 5.51)	-0.465	0.642
SUA (umol/L)	328.50 (281.00,383.23)	349.4 (291.90, 410.90)	-4.512	0.000
Glucose (mmol/L)	4.89 (4.46,5.31)	7.430 (6.110,9.840)	-23.748	0.000
Inflammatory index				
SII	353.60 (252.67,417.92)	444.56 (329.62,619.12)	-10.657	0.000
SIRI	0.555 (0.417,0.825)	0.756 (0.532,1.142)	-8.571	0.000
MHR	0.265 (0.209,0.384)	0.365 (0.265,0.505)	-10.578	0.000
UHR	244.35 (197.09,344.65)	345.11 (255.05,449.29)	-11.090	0.000

Data are expressed as n (%) for categorical variables and as M (Q3-Q1) for continuous variables with non-normal distribution. M (Q3-Q1), median (interquartile range); WBC, White blood cell; MONO, Monocyte; LYMP, Lymphocyte; PLT, Platelet; NEUT, Neutrophil; TC, Total cholesterol; LDL-C, Low-density lipoprotein cholesterol; TG, Triglyceride; HDL-C, High density lipoprotein cholesterol; SUA, serum uric acid; SII, systemic immune-inflammation index; SIRI, systemic inflammation response index; MHR, monocyte-to-high-density lipoprotein ratio; UHR, serum uric acid to HDL-C ratio. *P*<0.05 was considered statistically significant.

#### 3.1.2 Comparison of clinical characteristics and indicators of T2DM patients with different HOMA-IR levels

779 patients with T2DM were divided into three groups according to HOMA-IR. The average ages of the three groups of patients were 56(46.50,62.00) years old, 56(46.00,65.00) years old and 55 (44.00,63.00) years old respectively. The proportions of males in each group were 68.05%, 68.82% and 68.75% respectively. There was no statistically significant difference in age, gender, BMI, smoking and drinking among the three groups of patients (*P* > 0.05). There was no statistically significant difference in total cholesterol among the three groups of patients (*P* > 0.05). There were statistically significant differences in WBC, uric acid, fasting C-peptide, fasting insulin, Glucose, MONO, NEUT, PLT, LDL, triglycerides of TG, and total cholesterol of TC among the three groups of patients, and they showed an increasing trend with the increase of HOMA-IR score (*P*<0.05). There were statistically significant differences in the values of lymphocytes and HDL-C among the three groups of patients (*P*<0.05), and with the increase of HOMA-IR score, the values of lymphocytes and HDL-C showed a downward trend (*P*<0.05). The inflammatory indicators SII, SIRI, MHR and UHR of the three groups of patients were compared, and the differences were statistically significant. Moreover, with the increase of HOMA-IR score, all four inflammatory indicators showed an upward trend (*P*<0.05, **Table 2**).

**Table 2:** Comparison of clinical features of T2DM patients with different HOMA-IR scores [M (Q3–Q1)].

Characteristics	T2DM (n=779)			*χ^2^/F/Z*	*P*
	IS (n=385)	EIR (n=170)	SIR (n=224)		
Male, n (%)	262 (68.05%)	117 (68.82%)	154 (68.75%)	0.048	0.976
Age (years)	56 (46.50,62.00)	56 (46.00,65.00)	55 (44.00,63.00)	2.631	0.268
BMI (kg/m^2^)	24.14 (21.60, 27.67)	24.35 (21.58,27.80)	24.43 (21.65, 28.25)	1.733	0.178
Smoking, n (%)	156 (40.52%)	70 (41.18%)	91 (40.63%)	0.022	0.989
Drinking, n (%)	63 (16.36%)	26 (15.29%)	35 (15.63%)	0.121	0.941
Blood routine examination					
WBC (×109/L)	5.79 (4.69,6.93)	5.77 (4.88,6.81)	6.25 (5.32,7.61)^ab^	19.380	**0.000**
MONO (×10^9^/L)	0.34 (0.29,0.45)	0.37 (0.30,0.45)	0.39 (0.31,0.48)^a^	9.841	**0.007**
LYMP (×10^9^/L)	1.72 (1.39,2.11)	1.64 (1.42,2.06)	1.52 (1.28,1.92)^ab^	19.054	**0.000**
NEUT (×10^9^/L)	3.40 (2.70,4.32)	3.51 (2.86,4.45)^a^	3.82 (2.96,4.79)^ab^	16.044	**0.000**
PLT (×10^9^/L)	204 (177.5,231)	213.5 (185,241)^a^	234.5 (196.25,265.00)^ab^	46.656	**0.000**
Biochemical indicators					
LDL-C (mmol/L)	2.48 (1.81,3.06)	2.67 (1.90,3.26)^a^	2.72 (2.07,3.41)^b^	8.614	**0.013**
HDL-C (mmol/L)	1.09 (0.88,1.32)	0.96 (0.80,1.14)^a^	0.95 (0.82,1.11)^a^	40.985	**0.000**
TG (mmol/L)	1.26 (0.96,1.98)	1.84 (1.31,2.71)^a^	1.91 (1.38,3.00)^a^	74.477	**0.000**
TC (mmol/L)	4.69 (3.81,5.49)	4.51 (3.73,5.31)	4.77 (4.07,5.74)^b^	4.498	0.106
SUA (×10^9^/L)	311.00 (259.00,375.95)	355.45 (319.63,400.93)^a^	396.45 (344.68,452.15)^ab^	123.845	**0.000**
fasting C-peptide	0.87 (0.52,1.12)	1.59 (1.21,1.88)^a^	2.26 (1.72,2.94)^ab^	433.702	**0.000**
fasting insulin	3.00 (1.81,4.26)	7.00 (5.19,8.34)^a^	11.28 (8.75,15.27)^ab^	536.771	**0.000**
Glucose (mmol/L)	7.24 (5.86,9.47)	7.62 (6.14,9.74)	8.27 (6.83,10.87)^ab^	24.116	**0.000**
Inflammatory index					
SII	392.41 (302.84,545.09)	428.05 (331.34,598.32)^a^	561.95 (422.78,776.83)^ab^	66.152	**0.000**
SIRI	0.68 (0.49,1.02)	0.75 (0.55,1.05)	0.95 (0.61,1.36)^ab^	39.306	**0.000**
MHR	0.34 (0.23,0.45)	0.40 (0.30,0.51)^a^	0.40 (0.29,0.57)^a^	32.923	**0.000**
UHR	283.74 (204.38,388.59)	376.30 (294.77,461.87)^a^	412.19 (323.44,500.22)^ab^	110.592	0.000

Data are expressed as n (%) for categorical variables and as M (Q3-Q1) for continuous variables with non-normal distribution. M (Q3-Q1), median (interquartile range); BMI, Body mass index; WBC, White blood cell; MONO, Monocyte; LYMP, Lymphocyte; PLT, Platelet; NEUT, Neutrophil; TC, Total cholesterol; LDL-C, Low-density lipoprotein cholesterol; TG, Triglyceride; HDL-C, High density lipoprotein cholesterol; SUA, serum uric acid; SII, systemic immune-inflammation index; SIRI, systemic inflammation response index; MHR, monocyte-to-high-density lipoprotein ratio; UHR, serum uric acid to HDL-C ratio. *P*<0.05 was considered statistically significant.

a *P*<0.05 compared to the IS group.

b *P*<0.05 compared the EIR group.

P values shown in bold indicate that the statistical analysis is significant.

### 3.2 Correlation analysis

The correlation analysis results of serum SII, SIRI, MHR and UHR in patients with T2DM and HOMA-IR are shown in **Table 3**. Serum SII, SIRI, MHR and UHR in patients with T2DM were all positively correlated with HOMA-IR (*P* < 0.05). The rank correlation coefficients of SII, SIRI, MHR and UHR with HOMA-IR were 0.221, 0.170, 0.204 and 0.383, respectively.

**Table 3:** Correlation analysis of serum SII, MHR, cTnI, and Gensini scores in patients with AMI.

Indicators	HOMA-IR	
	*r_s_*	*P*
SII	0.221	0.000
SIRI	0.170	0.000
MHR	0.204	0.000
UHR	0.383	0.000

SII is the systemic immune-inflammation index; SIRI is the systemic inflammation response index; MHR is the ratio of monocytes to high-density lipoprotein cholesterol. UHR is the ratio of serum uric acid to high-density lipoprotein cholesterol; HOMA-IR is the homeostatic model assessment of IR.

### 3.3 Multivariate ordered regression analysis

The results of the parallelism hypothesis test showed that *P*=0.461 (>0.1), which was in line with the proportional dominance hypothesis, indicating that the regression equations of each model were parallel and meeting the premise of ordered logistic regression analysis. The influencing factors related to the degree of IR in the univariate analysis were included in the multivariate ordered logistic regression model (with the degree of IR as the dependent variable). The results showed that SII, SIRI, MHR and UHR were independent risk factors for IR in patients with T2DM (*P* < 0.05). The goodness of fit test results of the model (*χ²* = 1562.475, *P* = 0.013) indicated that the model fit well (**Table 4**).

**Table 4:** Multi-factor ordinal logistic regression for IR in patients with T2DM.

Indicators	β	Standard error	Wald χ^2^	Odds ratio	95% CI	*P*
SII	1.235	0.618	3.994	3.438	1.024-11.554	0.046
SIRI	1.403	0.574	5.967	4.067	1.319-12.528	0.015
MHR	1.025	0.652	2.877	2.787	1.809-10.004	0.027
UHR	1.480	0.983	3.273	4.393	2.585-29.488	0.001

SII is the systemic immune-inflammation index; SIRI is the systemic inflammation response index; MHR is the ratio of monocytes to high-density lipoprotein cholesterol. UHR is the ratio of serum uric acid to high-density lipoprotein cholesterol.

### 3.4 ROC curve analysis

**Figure 1:**
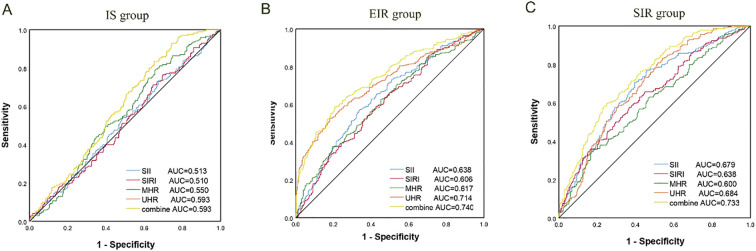
ROC curve for degree of IR in T2DM. **(A)** IS group; **(B)** EIR group; **(C)** SIR group. AUC, Area under the curve; SII, systemic immune-inflammation index; SIRI, systemic inflammation response index; MHR, monocyte-to-high-density lipoprotein ratio; UHR is the ratio of serum uric acid to high-density lipoprotein cholesterol.

In this study, the ROC curve was used to evaluate the discriminatory ability of novel inflammatory markers (SII, SIRI, MHR, UHR) for the degree of IR in patients with T2DM. The results showed that in the EIR group, the AUC values of SII, SIRI, MHR and UHR were 0.638, 0.606, 0.617 and 0.71 respectively (*P*<0.05). When the four indicators were combined for detection, the AUC increased to 0.740, and the sensitivity and specificity reached 58.73% and 79.24% respectively. For the SIR Group, the AUC values of each index were 0.679 for SII, 0.638 for SIRI, 0.600 for MHR, and 0.684 for UHR (*P*<0.05). After combined detection, the AUC increased to 0.733, at which point the specificity was 76.63% and the sensitivity was 58.03%. It IS worth noting that the AUC values of MHR and UHR in the IS group were 0.550 and 0.593 respectively (*P*<0.05); SII and SIRI did not show statistically significant in the IS group (*P* > 0.05). Studies have shown that the combined detection of SII, SIRI, MHR and UHR can be used as potential clinical utility indicators for evaluating the degree of IR in T2DM (for details, see **Table 5**; **Figure 1**).

**Table 5:** ROC curve analysis for the degree of IR in T2DM.

Group	Indicators	Sensitivity (%)	Specificity (%)	Cut-off point	AUC	95% CI	*P*
IS	SII	73.53	32.44	567.88	0.513	0.464-0.561	0.614
	SIRI	76.49	30.34	0.93	0.510	0.462-0.558	0.683
	MHR	79.42	34.03	0.29	0.550	0.504-0.596	0.045
	UHR	92.93	25.49	241.14	0.593	0.549-.637	0.000
	Comebine	92.94	25.54	0.18	0.593	0.549-0.637	0.000
EIR	SII	57.41	66.00	421.58	0.638	0.599-0.677	0.000
	SIRI	66.24	50.32	0.87	0.606	0.567-0.646	0.004
	MHR	37.73	80.54	0.28	0.617	0.577-0.656	0.000
	UHR	52.74	81.72	289.58	0.714	0.677-0.750	0.000
	Comebine	58.73	79.24	1.74	0.740	0.705-0.774	0.000
SIR	SII	70.50	60.44	448.93	0.679	0.638-0.720	0.000
	SIRI	57.62	63.21	0.85	0.638	0.595-0.681	0.000
	MHR	34.41	83.12	0.52	0.600	0.556-0.644	0.000
	UHR	48.33	80.81	309.22	0.684	0.646-0.723	0.000
	Comebine	58.03	76.63	0.31	0.733	0.696-0.770	0.000

SII is the systemic immune-inflammation index; SIRI is the systemic inflammation response index; MHR is the ratio of monocytes to high-density lipoprotein cholesterol. UHR is the ratio of serum uric acid to high-density lipoprotein cholesterol.

## 4 Discussion

T2DM is an urgent issue facing the health of the global population. The low treatment rate and compliance rate of diabetic patients also aggravate the occurrence and progression of chronic complications in diabetic patients. IR and/or islet β -cell dysfunction are the main causes of T2DM. Patients with T2DM have a long-term hyperglycemic state in their bodies, which is also prone to cause congenital and adaptive immune responses in the body, thereby leading to a chronic and low-grade inflammatory state of the body (30). In addition, the state of hyperglycemia and hyperlipidemia will further aggravate the damage of the islets and cause IR (31). The results of this study show that compared with the healthy population, the counts of WBC and its subcellular populations in patients with T2DM are significantly increased, and the levels of blood lipid, uric acid and blood glucose are also significantly increased. This is consistent with previous studies (31, 32). In addition, the levels of SII, SIRI, UHR and MHR in patients of the T2DM group were also significantly increased. It is indicated that the body of patients with T2DM is in a chronic and low-grade inflammatory state. If this state is maintained for a long time, it may promote the development of T2DM and lead to the occurrence of complications.

The inflammatory process plays an important role in the pathogenesis of T2DM, and the persistent chronic inflammatory response can lead to a decrease in the body’s sensitivity to insulin, causing IR. Therefore, it is very important to explore the role of new inflammatory markers in the occurrence and development of T2DM. Neutrophils are one of the important subgroups of white blood cells and have been proven to play a significant role in the inflammatory response of the body (33). When inflammation occurs in the body, it is the immune cell that responds first in the body, assists macrophages in aggregation, and interacts with antigen-presenting cells at the same time, further promoting the chronic inflammatory response (34). Platelets, known as “inflammatory cells”, are important indicators in routine blood cell counts. When activated, they adhere to endothelial cells and white blood cells and play a key role in inducing inflammatory responses by releasing pro-inflammatory compounds (35). Lymphocytes, as a part of adaptive immunity, play an important role in innate immunity and act as inflammatory mediators with regulatory and protective functions (36). Monocytes are a relatively special subgroup of white blood cells, which can differentiate into macrophages. Both have the ability to regulate inflammatory cytokines (37). Uric acid is synthesized by xanthine oxidase during purine metabolism and is also an extracellular antioxidant that can prevent oxidative stress. Normal uric acid levels have antioxidant effects, while high uric acid levels promote oxidation (38). In addition, abnormal lipid metabolism also plays an important role in the pathogenesis of T2DM and is an important risk factor for the development of T2DM. Studies have shown that hyperlipidemia can accelerate glucose-induced mitochondrial damage, thereby accelerating the occurrence and development of T2DM (32, 39). However, HDL-C has exactly the opposite effect and has antioxidant and anti-inflammatory effects in the occurrence and development of T2DM (40). The results of this study show that compared with patients sensitive to insulin, after IR occurs in patients with T2DM, neutrophils, monocytes, lymphocytes and platelets all increase. At the same time, their uric acid levels also increase significantly, while the HDL-C level decreases significantly. It is suggested that the levels of neutrophils, monocytes, lymphocytes, platelets, uric acid and HDL-C may play important biological roles in the occurrence and development of T2DM. However, due to individualized differences, when IR occurs in patients with T2DM, the above indicators may still be within the 95% confidence interval. Therefore, in recent years, new inflammatory markers based on blood cell subsets and biochemical indicators (such as HDL-C, SUA, etc.) have emerged, providing new and more comprehensive research directions for medical researchers.

SII is a new indicator for evaluating inflammation based on neutrophils, platelets, lymphocytes, etc., which can more objectively reflect the inflammatory changes of the body. SIRI combines neutrophils, monocytes and lymphocytes, etc., and is a novel and easily accessible biomarker of inflammation and the immune system, which is usually related to the intensity of the inflammatory response. Research shows (41) that there is a correlation between leukocytosis and chronic complications of diabetes, and the increase in white blood cells mainly reflects the elevation of neutrophils in the body. When inflammation occurs in the body, white blood cells respond rapidly to inflammatory stimuli, resulting in an increase in neutrophils in the circulation (42). In addition, the increase in interleukin levels can promote the reduction of lymphocytes and the increase of neutrophils (43). In patients with diabetes, platelets exhibit higher activity, leading to the release of inflammatory mediators and thereby attracting more platelets and WBCs to the inflammatory site (44). The MCP-1 secreted by monocytes and macrophages can promote the aggregation of inflammatory cells at the lesion site, thereby stimulating monocytes to secrete IL-1 and IL-6, putting the pancreatic tissue in a micro-inflammatory state, damaging endothelial cells, increasing blood glucose, generating oxidative stress, and triggering IR (45). UHR, which is the result of the combination of uric acid and HDL-C, is a sign of an increased inflammatory response in the body. MHR combines blood cell subsets (monocytes) with HDL-C and is an indicator for evaluating inflammation and oxidative stress. Juraschek et al. pointed out that hyperuricemia increases the risk of T2DM by 1.87 times and IR by 1.36 times (46). Furthermore, for every 1 mg/dL increase in serum uric acid level, the risk of T2DM increases by 17% (47). Furthermore, HDL-C is an anti-inflammatory and antioxidant factor, and the increase in its level is regarded as a protective factor against IR (48, 49). Therefore, SII, SIRI, UHR and MHR may be direct indicators of factors such as blood cell subcomponents, uric acid and HDL-C involved in the chronic inflammatory response of the body. Guo et al. (50) and Zhao et al. (51) conducted a study using NHANES data, and the results showed that there was a positive correlation between SII and HOMA-IR. The research results of Song et al. (26) show that the levels of SIRI were higher in T2DM-PAD patients, and they were independently linked with its clinical severity. In addition, both MHR (49) and UHR (52) are associated with IR levels. This study grouped patients with T2DM based on their different IR levels and observed the levels of SII, SIRI, UHR and MHR, which differed from the strategies of the above-mentioned studies. The results of this study show that the levels of SII, SIRI, UHR and MHR in patients of the T2DM group were significantly increased. Moreover, compared with patients sensitive to insulin, patients with early IR and those with significantIR have higher levels of SII, SIRI, UHR and MHR. Meanwhile, all four indicators were positively correlated with IR in patients with T2DM. Through multivariate ordered regression analysis, the results showed that all four indicators were independent risk factors for IR in patients with T2DM. In addition, the ROC analysis showed that the four indicators had good diagnostic efficacy for IR in patients with T2DM. However, in the IS group, SII and SIRI did not show statistically significant clinical utility. This might be because the bodies of insulin-sensitive individuals did not have obvious inflammatory responses, even though they were in a chronic and low-grade inflammatory state.

There is a potential mechanistic explanation for the association between SII, SIRI, and IR. Platelets play a central role in hemostasis and thrombosis and actively contribute to inflammatory responses by releasing various pro-inflammatory cytokines, growth factors, and chemokines. These mediators promote endothelial dysfunction, IR, and atherosclerosis (53). Neutrophils further propagate the inflammatory cascade by secreting pro-inflammatory cytokines, generating oxidative stress, and releasing proteolytic enzymes. These actions contribute to endothelial dysfunction, exacerbate IR, and induce pancreatic β-cell apoptosis (54). Monocytes and macrophages secrete monocyte chemoattractant protein-1 (MCP-1), which facilitates the recruitment of inflammatory cells to sites of injury. This process stimulates monocytes to release interleukin (IL)-1 and IL-6, resulting in a persistent low-grade inflammatory state within pancreatic tissue. This inflammation damages endothelial cells, elevates blood glucose levels, induces oxidative stress, and ultimately triggers IR (45). Lymphocytes, as key mediators of adaptive immunity, upon activation, release cytokines that amplify systemic inflammation, disrupt glucose homeostasis, and accelerate the progression of diabetic complications (55). In T2DM, elevated levels of neutrophils, monocytes, and platelets, along with relative lymphopenia, are commonly observed, underscoring the pivotal role of chronic inflammation in disease onset and progression. These alterations contribute to increased levels of SII and SIRI, reflecting a heightened inflammatory state that mediates IR. Potential mechanisms linking MHR and UHR with insulin resistance have also been proposed. Recent experimental studies have confirmed the critical involvement of monocyte-derived immunity in the pathogenesis of T2DM, contributing to β-cell dysfunction, impaired insulin secretion, and the development of IR (56). Elevated uric acid levels can enhance adipocyte oxidative stress by upregulating MCP-1 expression and downregulating adiponectin, a pro-oxidative effect that may promote adipose tissue accumulation, thereby contributing to insulin resistance (57). Additionally, uric acid-induced reductions in nitric oxide bioavailability impair skeletal muscle glucose uptake, further aggravating IR (52, 58). HDL-C exerts multiple protective effects, including reverse cholesterol transport, anti-inflammatory actions, antithrombotic properties, vasodilation, and anti-apoptotic functions (59). In patients with T2DM, monocyte counts and blood uric acid levels are elevated, while HDL-C levels are reduced. These changes lead to increased levels of the MHR and UHR, which may promote the development of insulin resistance. Given that SII, SIRI, MHR, and UHR are composite markers reflecting both inflammatory activity and lipid metabolism, our findings suggest that these four indices may serve as potential biomarkers for identifying insulin resistance in individuals with T2DM.

To sum up, the levels of the novel inflammatory markers SIRI, SII, UHR and MHR in patients with T2DM increase and are positively correlated with IR. They are independent risk factors for IR in T2DM patients and have clinical utility to a certain extent, which can provide a reference basis for the early clinical prevention, diagnosis and treatment of IR in T2DM patients. This study also has certain limitations. Although the four inflammatory indicators are readily available in clinical practice, due to individual differences, the reduction in the numbers of lymphocytes, neutrophils or platelets is also very common and may lead to selection bias. In addition, some unknown confounding factors, such as the duration of T2DM, have an impact on the research conclusions. And, the retrospective study also limited the persuasiveness of the results of this study and prevented a comprehensive observation of the dynamic changes of each index. Although the AUC of the combined detection of the EIR and SIR Groups did not reach the high accuracy (AUC > 0.9) in the results of this study, it was still superior to the detection of a single indicator. This indicates that large-sample, multi-center prospective studies are still needed before actual clinical application to provide theoretical support for this purpose, which also provides a direction for subsequent researchers. In the future, we will also expand the sample size and improve the research methods, with the aim of providing a more comprehensive reference basis for the clinical diagnosis and treatment of IR in patients with T2DM.

## Data Availability

The raw data supporting the conclusions of this article will be made available by the authors, without undue reservation.
